# Knee Load Distribution in Hip Osteoarthritis Patients After Total Hip Replacement

**DOI:** 10.3389/fbioe.2020.578030

**Published:** 2020-09-21

**Authors:** Stefan van Drongelen, Mariska Wesseling, Jana Holder, Andrea Meurer, Felix Stief

**Affiliations:** ^1^Dr. Rolf M. Schwiete Research Unit for Osteoarthritis, Orthopaedic University Hospital Friedrichsheim gGmbH, Frankfurt, Germany; ^2^Human Movement Biomechanics Research Group, Department of Movement Sciences, KU Leuven, Leuven, Belgium; ^3^Orthopaedic University Hospital Friedrichsheim gGmbH, Frankfurt, Germany; ^4^Goethe University Frankfurt, Frankfurt, Germany

**Keywords:** joint contact forces, musculoskeletal modeling, total hip replacement, knee adduction moment, walking, osteoarthritis

## Abstract

Reduced external knee adduction moments in the second half of stance after total hip replacement have been reported in hip osteoarthritis patients. This reduction is thought to shift the load from the medial to the lateral knee compartment and as such increase the risk for knee osteoarthritis. The knee adduction moment is a surrogate for the load distribution between the medial and lateral compartments of the knee and not a valid measure for the tibiofemoral contact forces which are the result of externally applied forces and muscle forces. The purpose of this study was to investigate whether the distribution of the tibiofemoral contact forces over the knee compartments in unilateral hip osteoarthritis patients 1 year after receiving a primary total hip replacement differs from healthy controls. Musculoskeletal modeling on gait was performed in OpenSim using the detailed knee model of Lerner et al. (2015) for 19 patients as well as for 15 healthy controls of similar age. Knee adduction moments were calculated by the inverse dynamics analysis, medial and lateral tibiofemoral contact forces with the joint reaction force analysis. Moments and contact forces of patients and controls were compared using Statistical Parametric Mapping two-sample *t*-tests. Knee adduction moments and medial tibiofemoral contact forces of both the ipsi- and contralateral leg were not significantly different compared to healthy controls. The contralateral leg showed 14% higher medial tibiofemoral contact forces compared to the ipsilateral (operated) leg during the second half of stance. During the first half of stance, the lateral tibiofemoral contact force of the contralateral leg was 39% lower and the ratio 32% lower compared to healthy controls. In contrast, during the second half of stance the forces were significantly higher (39 and 26%, respectively) compared to healthy controls. The higher ratio indicates a changed distribution whereas the increased lateral tibiofemoral contact forces indicate a higher lateral knee joint loading in the contralateral leg in OA patients after total hip replacement (THR). Musculoskeletal modeling using a detailed knee model can be useful to detect differences in the load distribution between the medial and lateral knee compartment which cannot be verified with the knee adduction moment.

## Introduction

Hip osteoarthritis (OA) is one of the most common degenerative diseases of the musculoskeletal system (Fuchs et al., 2013). Patients, who already had a unilateral total hip replacement (THR) for end-stage hip OA, have an increased risk of developing OA in other joints of their lower extremities (Shakoor et al., 2002).

Stief et al. (2018) showed that after THR, patients reduce the external knee adduction moment (KAM) in the second half of stance of both legs compared to healthy controls whereas Shakoor et al. (2003) found a significantly higher KAM in the contralateral knee. The reduction in KAM is thought to shift the load from the medial to the lateral knee compartment (Schmidt et al., 2017). An increased probability of lateral knee OA would support the findings of Weidow et al. (2005), who showed that lateral OA of the knee was more frequently associated with hip OA on the ipsilateral side than medial knee OA.

KAM is considered to be only a surrogate for the load distribution between the medial and lateral compartments of the knee and not a valid measure for the tibiofemoral contact forces (CF) which are the result of externally applied forces and muscle forces (Winby et al., 2009). Joint CF, calculated by musculoskeletal modeling, have been validated by direct (*in vivo*) measurements in patients with instrumented implants (Fregly et al., 2012). An increased KAM is related to higher medial tibiofemoral contact forces (MKCF) during normal gait (Kutzner et al., 2013). However, the relation is not always straightforward as various gait retraining programs, with the intention to reduce KAM, found similar MKCF as in normal gait (Walter et al., 2010; Pizzolato et al., 2017). In addition, Pizzolato et al. (2017) reported that reduced MKCF are not inevitably associated with increased lateral tibiofemoral contact forces (LKCF).

Most studies investigating joint CF of patients receiving THR and the progression of these forces after THR, reported on hip CF (Meyer et al., 2018; O’Connor et al., 2018), whereas only few studies also examined knee CF in these patients (Shakoor et al., 2003; Wesseling et al., 2018). Shakoor et al. (2003) reported increased reaction forces (not including the muscle activation), in the medial knee compartment of the contralateral leg compared to the operated leg. When muscle forces are not included in the calculation of joint load, this may lead to an underestimation of the actual load in the joint (Lloyd and Buchanan, 2001). Several studies already showed a contribution of muscular co-contraction to higher joint CF (Trepczynski et al., 2018; Hoang et al., 2019). Wesseling et al. (2018) reported that 12 months after THR surgery the total CF on the ipsilateral knee were still lower compared to healthy controls. Since Wesseling et al. (2018) focused on the total knee CF, they could not provide information on the load on the medial and lateral compartments. It is known that after THR, hip OA patients have a higher risk for the development of OA in the contralateral knee joint (Jungmann et al., 2015; Joseph et al., 2016), however, these studies did not specify the medial or lateral knee compartment. Information on a possible asymmetry or imbalance across the medial and lateral compartments is important with regard to the development of OA: a non-physiological knee loading can precipitate OA in a healthy joint (Andriacchi et al., 2004, 2009).

In the present study the MKCF and LKCF are studied independently, using a more detailed knee model introduced by Lerner et al. (2015). Consequently, the purpose of the present study was to investigate tibiofemoral CF in the knees of unilateral hip THR patients after THR surgery separately for the medial and lateral compartment. This study hypothesized that MKCF and LKCF are different in patients with unilateral hip OA after THR surgery compared to healthy controls in such a way that the lateral knee compartment is loaded more and the medial knee compartment is loaded less.

## Materials and Methods

### Participants

Nineteen patients, scheduled for THR due to unilateral hip OA, were included in this study (Table 1). Exclusion criteria were: the inability to walk without walking aids, Body Mass Index (BMI) above 30, inflammatory arthritis, orthopedic surgeries within the past 6 months, OA in lower limb joints other than the affected hip, and previous lower extremity joint replacement. All surgeries were performed, using a lateral approach, by experienced orthopedic surgeons.

**TABLE 1:** Anthropometric data and walking speed of patients and healthy controls.

	postoperative patients (*N* = 19)	healthy controls (*N* = 15)	*p*-value
Age (years)	65.5 (7.9)	61.1 (8.3)	0.126
Height (m)	1.70 (0.07)	1.72 (0.08)	0.377
Body mass (kg)	75.7 (10.9)	67.8 (10.3)	**0.041**
Body Mass Index (kgm^–2^)^§^	27.4 (23.7–28.1)	24.1 (20.4–24.6)	**<0.001**
Gender	10 female/9 male	6 female/9 male	0.464
Walking speed (ms^–1^)	1.18 (0.11)	1.25 (0.12)	0.072

*Mean (standard deviation) for subject characteristics and walking speed. ^§^ Not normally distributed in healthy controls; median and (interquartile range) provided. Significant differences are bold printed.*

Patient gait data were compared to data of 15 healthy controls with similar age distribution (Stief et al., 2018). Healthy subjects were included if they had no history of orthopedic surgeries or chronic and neuromuscular disease. Our institution’s medical ethics committee approved the study (123/13 and 497/15) and all patients and healthy subjects gave informed consent prior to participation.

### Gait Analysis

For the patients, three-dimensional gait analysis was performed in the week before surgery and on average 16 (6) months postoperatively. All patients were asked to walk barefoot at a self-selected speed in the level gait laboratory. Kinematic data were collected using 8 Vicon MX T10 cameras (VICON Motion Systems, Oxford, United Kingdom) operating at 200 Hz. Additionally, two AMTI force plates (Advanced Mechanical Technology Inc., Watertown, MA, United States) were used to synchronously collect kinetic data at 1000 Hz.

A marker set (called MA) was used which includes, in addition to the standardized Plug-in-Gait marker set (Kadaba et al., 1990), reflective markers on the medial malleolus, medial femoral condyle and greater trochanter (Stief et al., 2013). An overview of all markers can be found in Supplementary Figure 1. A static standing trial was performed to be able to scale the musculoskeletal model. During the static upright standing trial, participants stood barefoot, feet shoulder width apart with the knees fully extended. Of all performed trials, three postoperative trials were processed per subject.

For the control subjects, the three-dimensional gait analysis was performed only once. First, the healthy controls walked at a self-selected speed, to be able to compare the patients’ postoperative data (Table 1). Further, to compare preoperative patients to healthy controls, healthy controls also walked at a slow walking speed of approximately 1.0 ms^–1^, comparable to patients shortly before surgery (Schmidt et al., 2017). In the control subjects, only one side (the left side was chosen randomly) was used for further analysis and for comparison with the ipsi- and contralateral side of the patient group.

### Musculoskeletal Modeling

**FIGURE 1:**
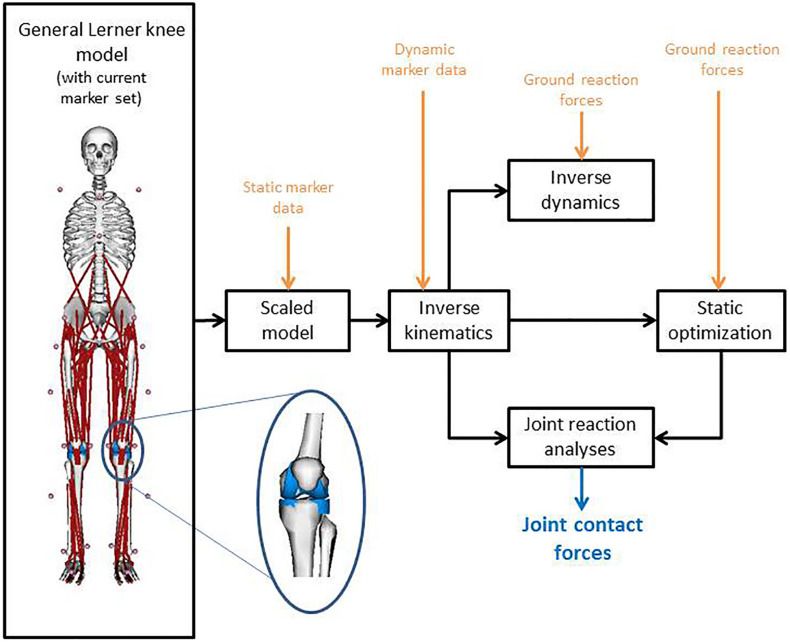
Model with the used marker set, combined with the workflow on implementing the motion capture data into OpenSim to calculate the joint contact forces. The inset graphic shows the medial and lateral compartment of the knee of the used musculoskeletal model according to Lerner et al. (2015).

Figure 1 shows the full-body musculoskeletal model with a detailed knee joint (Lerner et al., 2015) that was used to analyze the gait data in OpenSim 3.3 (Delp et al., 2007). The model is based on a previously described full body musculoskeletal model (Delp et al., 1990; Demers et al., 2014) and includes 18 body segments and 92 muscle-tendon actuators. Metatarsophalangeal and subtalar joints were fixed in anatomical neutral positions for all analyses (O’Connor et al., 2018). A fourth order zero-lag low-pass Butterworth filter with a cut-off frequency of 10 Hz was applied to the ground reaction forces, whereas a Woltring filter with a mean square error value of 10 was used to smooth the marker data (Woltring, 1991). Input for the model was created with the MOtoNMS toolbox (Mantoan et al., 2015) which processes the experimental data (3D marker positions and ground reaction forces) from C3D files. The joint centers of the hip, knee and ankle joint were calculated within the MOtoNMS toolbox (Mantoan et al., 2015). The hip joint center was calculated according to Harrington et al. (2007), while the knee and ankle joint centers were computed as the mid points between femoral condyle markers and the medial and lateral malleolus, respectively.

The model was scaled for each subject and for the preoperative and postoperative measurements separately, using the marker positions of the static standing trial and the measured body mass. Inverse kinematics and inverse dynamics were performed to calculate joint angles and moments, respectively. The knee adduction moment around the medial condyle (MKAM) was normalized to body mass and expressed in Nmkg^–1^. Muscle forces were calculated using a static optimization approach (Anderson and Pandy, 2001). The objective function for static optimization was to minimize the sum of squared muscle activations. In a final step, joint reaction analysis was performed to calculate the MKCF and LKCF (Figure 1). CF were normalized to body weight. All data were time-normalized to the stance phase of gait (101 time points, from heel strike to foot-off of the same foot, detected using the ground reaction forces with a threshold of 20N).

### Statistics

Statistical data analyses for the anthropometrics and gait speed were performed with SPSS (version 26, IBM Corporation, New York, NY, United States). Shapiro-Wilk tests were used to test for normal distribution of the above mentioned parameters. Unpaired Student’s *t*-tests were used to determine statistical differences between the anthropometrics of controls and patients in age, height, body mass and speed, whereas Mann-Whitney tests were used to determine statistical differences in BMI. Further, a χ^2^ test was used to compare gender distribution in the two groups.

Joint angles, MKAM, MKCF, LKCF and the ratio between the lateral and total CF were evaluated using Statistical Parametric Mapping (SPM). SPM is based on Random Field Theory (Adler and Taylor, 2007) and has been validated for 1D data (Pataky et al., 2013, 2016). All SPM analyses were implemented using the open-source spm1d code (version M.0.4.3^1^) in MATLAB. Controls and patients were compared using SPM two-sample *t*-tests, data of the ipsilateral and contralateral leg were compared with SPM paired sample *t*-tests. A critical threshold of α = 0.05 was used. When the waveform exceeded the critical threshold, the data were considered significantly different in that part of stance. Differences were considered significant when differences were found between more than four successive time points, i.e., at least 4% of the stance phase of the gait cycle, similar to Wesseling et al. (2018).

*Post hoc* power calculations and the effect size Cohens’s *d* were determined for the main findings using Gpower (Faul et al., 2007) and according to Cohen (2013). To express the percentage differences between groups as well as between legs, and as input for the power calculations the values and standard deviations at the time of the maximum difference between the curves (within the band of significant differences from the SPM) were extracted.

## Results

### Participants

Significant differences between postoperative patients and healthy controls were found in body mass and BMI (Table 1). No significant differences were found in gender distribution, age, height, and walking speed.

### Kinetics

**FIGURE 2:**
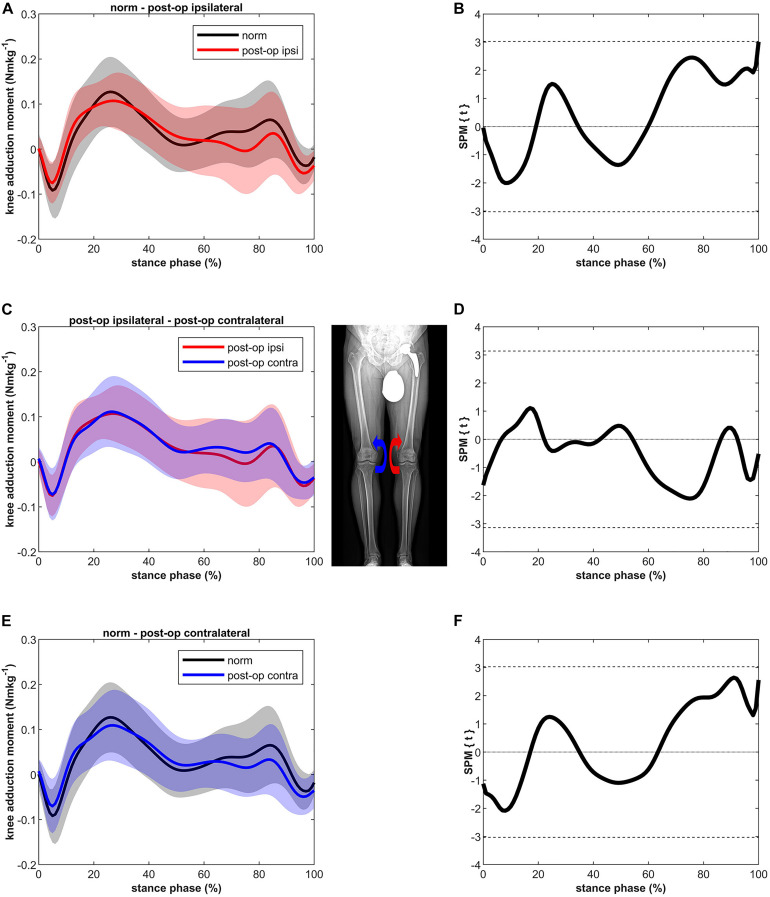
Curves of the external knee adduction moment (bands represent standard deviation) on the left side and the SPM *t*-test results on the right side; comparing controls (norm in black) to ipsilateral side of postoperative patients (post-op ipsi in red) at the top **(A,B)**, ipsilateral side of postoperative patients (post-op ipsi in red) to the contralateral side (post-op contra in blue) in the middle **(C,D)** and controls (norm in black) to the contralateral side of the postoperative patients (post-op contra in blue) at the bottom **(E,F)**. When the SPM *t*-values exceed the critical threshold (dashed horizontal line), differences are significant. Where significant differences are found for more than 4 successive time points (4% of the stance phase of the gait cycle), areas are shaded gray and *p*-values are reported. The knee adduction moments are normalized to body mass and expressed in Nmkg^–1^.

Although slightly lower in the second half of stance, MKAM of the ipsilateral and the contralateral leg after THR were not significantly different compared to MKAM of healthy controls (Figure 2). No differences were found between the ipsilateral and contralateral leg.

**FIGURE 3:**
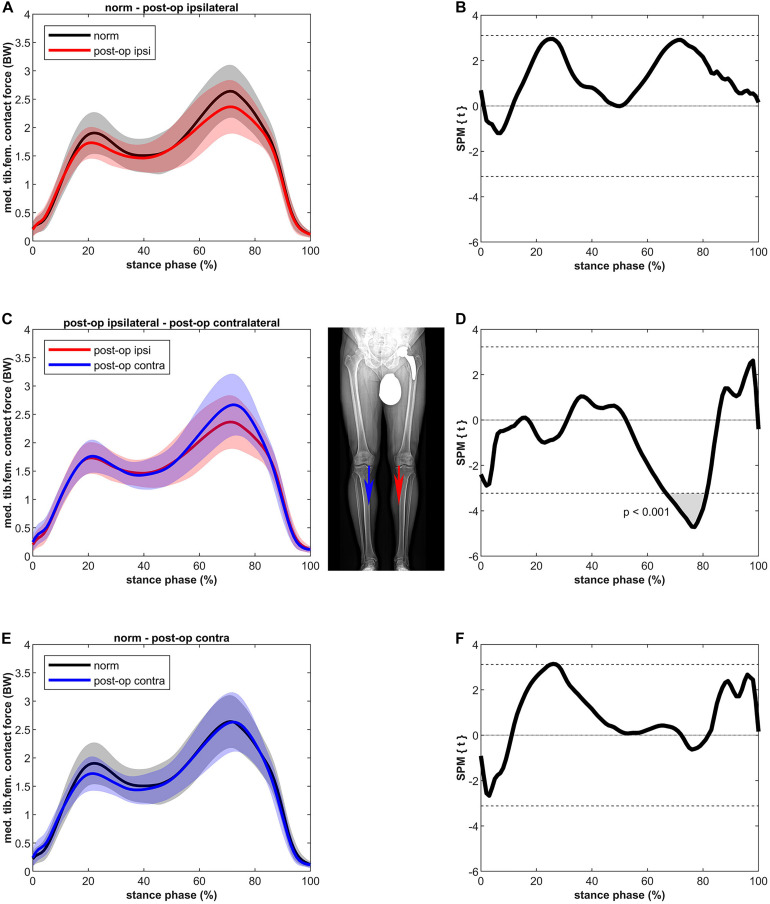
Curves of the tibiofemoral contact force on the medial epicondyle (bands represent standard deviation) on the left side and the SPM *t*-test results on the right side; comparing controls (norm in black) to ipsilateral side of postoperative patients (post-op ipsi in red) at the top **(A,B)**, ipsilateral side of postoperative patients (post-op ipsi in red) to the contralateral side (post-op contra in blue) in the middle **(C,D)** and controls (norm in black) to the contralateral side of the postoperative patients (post-op contra in blue) at the bottom **(E,F)**. When the SPM *t*-values exceed the critical threshold (dashed horizontal line), differences are significant. Where significant differences are found for more than 4 successive time points (4% of the stance phase of the gait cycle), areas are shaded gray and *p*-values are reported. Contact forces are normalized to body weight (BW).

The MKCF of the ipsilateral leg showed slightly lower values compared to the healthy controls in both the first and second half of stance, however, these differences were not significant (Figure 3). In contrast to the ipsilateral leg, the MKCF of the contralateral leg showed only lower values during the first half of stance, but also these differences did not reach a significant level. MKCF of the contralateral leg was higher (max 14%) compared to the ipsilateral leg in the second half of stance (*p* < 0.001, 67–81% of stance, power = 100%, effect size *d* = 3.66).

**FIGURE 4:**
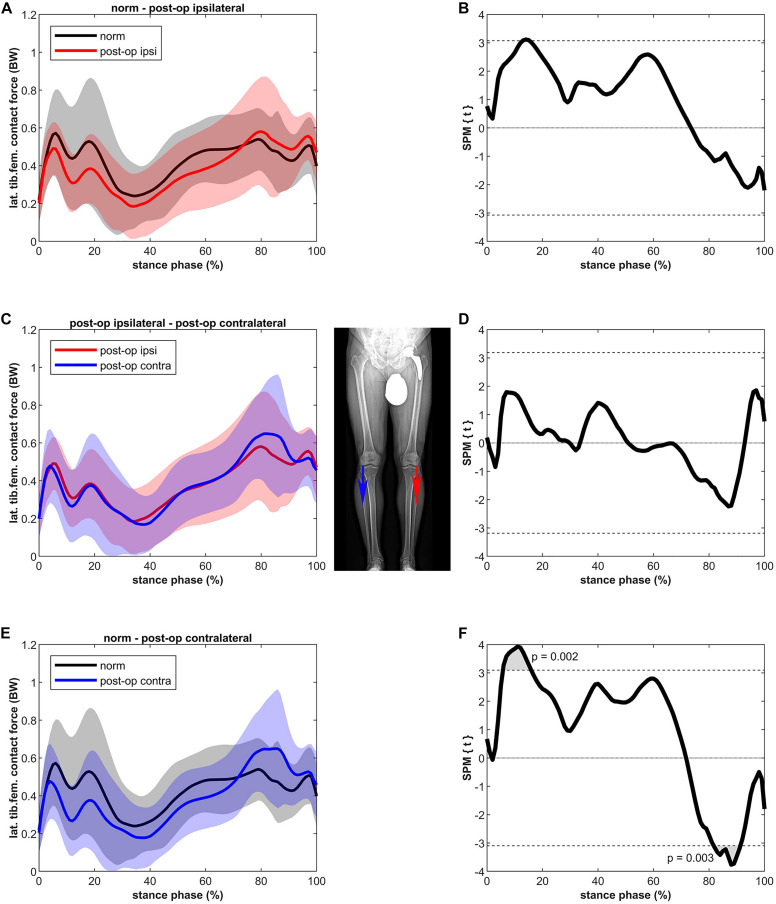
Curves of the tibiofemoral contact force on the lateral epicondyle (bands represent standard deviation) on the left side and the SPM *t*-test results on the right side; comparing controls (norm in black) to ipsilateral side of postoperative patients (post-op ipsi in red) at the top **(A,B)**, ipsilateral side of postoperative patients (post-op ipsi in red) to the contralateral side (post-op contra in blue) in the middle **(C,D)** and controls (norm in black) to the contralateral side of the postoperative patients (post-op contra in blue) at the bottom **(E,F)**. When the SPM *t*-values exceed the critical threshold (dashed horizontal line), differences are significant. Where significant differences are found for more than 4 successive time points (4% of the stance phase of the gait cycle), areas are shaded gray and *p*-values are reported. Contact forces are normalized to body weight (BW).

Figure 4 shows that the LKCF of the ipsilateral leg was not significantly different compared to healthy controls. The contralateral leg did not differ from the ipsilateral leg; however, some differences were detected compared to the healthy controls. The contralateral leg of the patients showed a lower LKCF (39%) between 6 and 16% of stance (*p* = 0.002, power = 67%, effect size *d* = 0.73) in relation to the healthy controls whereas the contralateral leg of the patients showed a significantly higher LKCF (39%) between 82 and 91% of stance (*p* = 0.003, power = 64%, effect size *d* = 0.70).

**FIGURE 5:**
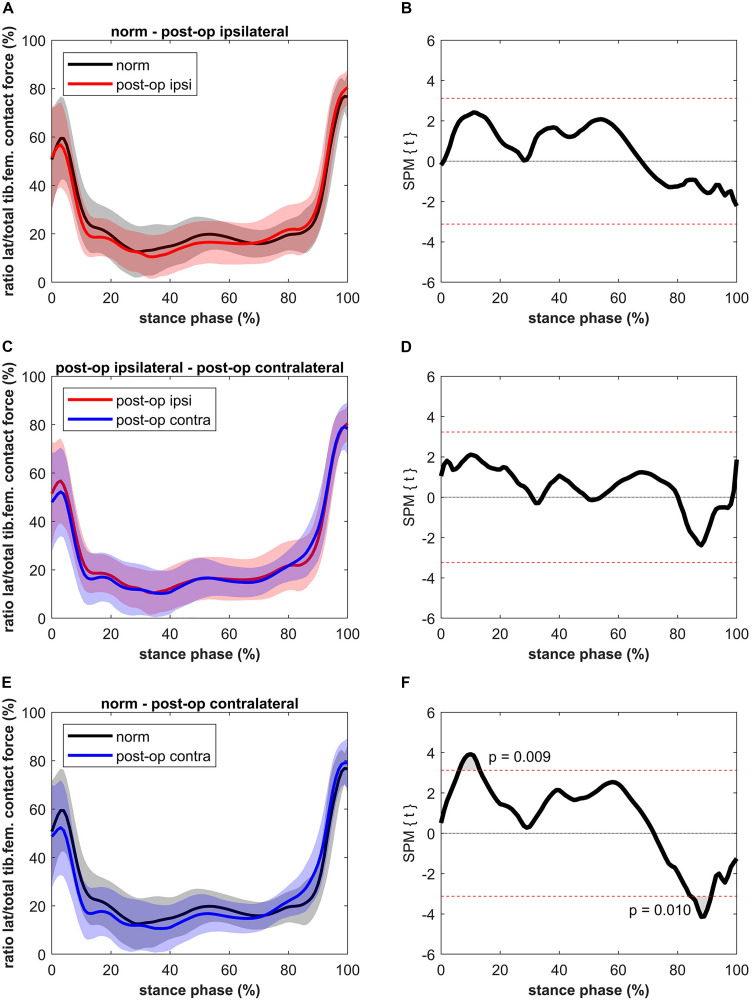
Curves of the ratio between the lateral tibiofemoral contact force and the total tibiofemoral contact force (bands represent standard deviation) on the left side and the SPM *t*-test results on the right side; comparing controls (norm in black) to ipsilateral side of postoperative patients (post-op ipsi in red) at the top **(A,B)**, ipsilateral side of postoperative patients (post-op ipsi in red) to the contralateral side (post-op contra in blue) in the middle **(C,D)** and controls (norm in black) to the contralateral side of the postoperative patients (post-op contra in blue) at the bottom **(E,F)**. When the SPM *t*-values exceed the critical threshold (dashed horizontal line), differences are significant. Where significant differences are found for more than 4 successive time points (4% of the stance phase of the gait cycle), areas are shaded gray and *p*-values are reported.

The ratio between the CF on the lateral condyle and the total CF for the ipsilateral leg was not significantly different compared to the healthy controls (Figure 5). Also, no differences were found compared to the contralateral leg. Short significant differences were detected when comparing the contralateral leg to the healthy controls. The healthy controls showed a higher ratio (max 32%) between 6 and 13% of stance (*p* = 0.009, power = 61%, effect size *d* = 0.68) and a lower ratio (max 26%) compared to the contralateral leg of the patients between 85 and 91% of stance (*p* = 0.010, power = 75%, effect size *d* = 0.82).

Figures with the results of the SPM analyses of the preoperative data were reported in the Supplementary Material (Supplementary Figures 2–4).

### Kinematics

The kinematics for the hip (flexion-extension and adduction-abduction) and the knee (flexion-extension) after THR were presented in Supplementary Material (Supplementary Figures 5–7).

For hip extension no differences were found compared to the healthy controls, but the ipsilateral leg showed significantly less extension in the second phase of stance compared to the contralateral leg (*p* < 0001, 54–100% of stance, Supplementary Figure 5).

Hip adduction was similar for the ipsilateral and contralateral leg, but significantly less compared to healthy controls during the first half of stance (18–40 and 16–58% of stance for the ipsilateral and contralateral leg, respectively, Supplementary Figure 6).

Knee flexion during the first half of stance equaled that of healthy controls and of the contralateral leg. The extent of knee extension in the ipsilateral leg was significantly lower compared to healthy controls (*p* < 0001, 61–100% of stance) and compared to the contralateral leg (*p* < 0001, 52–100% of stance, Supplementary Figure 7).

## Discussion

The goal of this research was to study tibiofemoral CF during gait in unilateral hip OA patients after THR. It has been proposed that a reduced KAM in the second half of the stance phase shifts the distribution of the knee joint CF from medial to lateral and therefore, increases the load on the lateral compartment (Schmidt et al., 2017). In the present study we found no differences in the MKCF compared to healthy controls for both the ipsi-and contralateral leg. In the ipsilateral leg no significant shift of the tibiofemoral CF from the medial to the lateral condyle after THR was detected, as the LKCF were nearly identical to healthy controls. Only in the contralateral leg a higher lateral knee joint loading in the second half of the stance phase was found.

The MKAM reported here agree with the KAM reported in the study of Stief et al. (2018) in which KAM in the second half of the stance was still lower in patients after THR compared to healthy controls. Stief et al. reported significant differences whereas in the present study the values were only slightly lower. An explanation for these discrepancies might be that different methods were used to calculate the external joint moments. Stief et al. (2013) used an adapted lower body protocol and calculated the joint moments around the joint centers directly from the gait analysis (Davis et al., 1991). In the present study, the external joint moments were calculated with the inverse dynamics tool of OpenSim, where KAM is calculated at the medial condyle, which means it is shifted medially relative to the joint center. Further, Stief et al. compared peak values whereas in the present study, the whole stance phase was compared by SPM.

Like MKAM, MKCF of the ipsi- and contralateral leg did not deviate significantly from healthy controls. All in all, our MKCF agree with that of Wesseling et al. (2018) who studied longitudinal total knee CF after THR. Specifically, the second peak of the ipsilateral leg remained lower than controls 1 year after surgery, and they concluded that no overloading was present 1 year after THR, neither in the ipsilateral nor the contralateral knee. In contrast to Wesseling et al. (2018), who used the generic musculoskeletal model of OpenSim, we used the model by Lerner et al. (2015) which allows detailed analysis of medial and lateral tibiofemoral CF.

For the ipsilateral leg, lateralization of the load was not found, as the LKCF were similar to healthy controls. For the contralateral leg the LKCF were lower during the first half of stance and higher during the second half of stance compared to healthy controls. The latter might indicate a shift of the tibiofemoral CF to the lateral compartment. It must be noted that the absolute differences are small and only for a short period (between 82 and 91% of stance). The differences as such might not be clinically relevant.

The higher LKCF for the contralateral leg resulted in a higher ratio of the lateral to total CF for a period of 6% in the second half of stance. In general, the distribution of the forces over the medial and lateral compartments (a clear higher medial to lateral loading) agrees with previous data (Lerner et al., 2015). A finite element study of Yang et al. (2010) showed that, as in the present study (Figure 4), approximately 80% of the tibiofemoral forces are distributed to the medial knee compartment and 20% to the lateral compartment. Yang et al. (2010) were even able to distinguish leg alignments as the force distributed to the lateral compartment was 19, 21, and 22% for the varus, normal and valgus aligned knee, respectively. The higher ratio at the beginning and at the end of the stance phase is due to the external knee abduction moment at heel strike and toe-off (Yang et al., 2010).

Changes in external hip and knee joint moments and joint CF are caused by changes in the kinematics (Wesseling et al., 2015). Deviating kinematics in the ipsilateral leg can persist even 1 year after THR (Foucher et al., 2007). In the present study, it was shown that hip adduction (in the first half of stance) and knee extension (in the second half of stance) were still less in postoperative patients compared to the healthy controls. Recently, it has been shown that differences in sagittal plane gait kinematics between patients with knee OA and asymptomatic controls appear to be mainly a result of reduced walking speed (Ismailidis et al., 2020). Walking speed can also influence joint CF (O’Connor et al., 2018). O’Connor et al. (2018) reported higher hip CF in THR patients during walking at higher speeds. However, for the knee CF less information is available, especially when considering MKCF and LKCF. Lenton et al. (2018) published on the effect of speed on medial and total tibiofemoral CF, whereas no effect on the lateral CF was found. However, the walking speeds were much higher (1.53 and 1.81 ms^–1^) so it must be questioned if the results can be transferred to the slower walking speed of patients, as measured in the present study. The strength of the present study is that CFs were compared between patients after THR and a healthy control group with an almost identical walking speed (no significant differences). Accordingly, our results cannot be related to differences in walking speed.

In the present study a significant difference in body mass and BMI was found between healthy controls and patients. However, we normalized the joint moments and joint CF to body mass/body weight, thus eliminating the effect of BMI and body mass on our main findings. Also, Harding et al. (2016) found significant OA effects in peak tibiofemoral compression forces when the forces were normalized to body mass. In addition, they found significant BMI effects only in the case of absolute, non-normalized forces. In the present study, and in the above cited study by Harding et al. (2016) subjects with nominal BMI were included: therefore it is possible that differences in tibiofemoral CF, greater than those which can be attributed to higher body mass are only valid for grossly obese individuals (Browning and Kram, 2007; Messier et al., 2014).

Higher CF on the contralateral (non-operated) side in the second half of stance compared to the ipsilateral side confirm the results of other studies (Shakoor et al., 2003; Foucher and Wimmer, 2012; Wesseling et al., 2018), although not all studies reported significant differences between patients (ipsilateral or contralateral) and healthy controls (Wesseling et al., 2018) so that a surplus load cannot be confirmed. The present results do support studies reporting a higher risk for the development of OA in the contralateral knee joint after THR (Shakoor et al., 2002; Umeda et al., 2009; Gillam et al., 2013; Jungmann et al., 2015; Joseph et al., 2016) since patients are walking with an asymmetrical limb load. However, these studies only report more structural damage and progression of degenerative findings in the contralateral knee and do not specify the medial or lateral knee compartment. Gillam et al. (2013) stated that further studies are needed to investigate whether an increased risk of receiving an arthroplasty in the contralateral knee is only related to having a hip arthroplasty or if other factors such as pain play a role as well. One factor which can be decisive is the leg alignment. Previous studies found that a valgus malalignment increases LKCF (Holder et al., 2020) and therefore might increase the risk to suffer lateral knee OA and lateral cartilage damage (Felson et al., 2013). The missing information on the leg alignment is a limitation of the present study. With that information the musculoskeletal model could have been optimized for the patient-specific tibiofemoral alignment. Previous research by Lerner et al. (2015) used data from anterior-posterior full-leg radiographs to show that MKCF and LKCF can accurately predict *in vivo* measurements. In the present study, these data were not available as in the clinical standard only a pelvic overview X-Ray was made to check for loosening of the prosthesis. We expect that the leg alignment has only a marginal effect on the results as previous work showed that the implantation of a hip prosthesis only led to a slight increased varus alignment (1°) of the operated leg (van Drongelen et al., 2019).

Another limitation of the present study is the relatively small sample size. *Post hoc* power analysis and the determination of the effect size for the primary findings, however, revealed that the sample size of 19 patients provided 100% power for the difference in the MKCF between the ipsilateral and contralateral leg. Further, the differences in the LKCF and the ratio between the LKCF and total CF between our study groups (19 patients, 15 controls) had a power between 61 and 75%. The calculated effect sizes between 0.68 and 3.66 observed for the main findings in the current study reinforces the relevance of the results. Nevertheless, some results of the present study may be underpowered and the differences could be significant with greater sample sizes.

## Conclusion

The hypothesis that the distribution of tibiofemoral CF is different in patients with unilateral hip OA patients after THR compared to healthy controls cannot be fully confirmed by this study. The CF on the medial tibiofemoral compartment for both the ipsi- and contralateral leg did not differ from healthy controls. Also, the CF on the lateral compartment of the ipsilateral leg was similar to controls. Only a higher LKCF and higher ratio between the LKCF and total CF in the contralateral leg indicates a higher lateral knee joint loading in hip OA patients after receiving a THR. Nevertheless, it must be noted that the differences found are only for a short period during the second half of stance. Though, musculoskeletal modeling can be useful to detect differences in the load distribution between the medial and lateral knee compartment, which cannot be verified with KAM. The present study cannot finally clarify whether this difference is sufficient to cause permanent damage to the lateral knee compartment in patients with unilateral hip OA after receiving a THR.

## Data Availability Statement

The raw data supporting the conclusions of this article will be made available by the authors, without undue reservation.

## Ethics Statement

The studies involving human participants were reviewed and approved by Medical Ethics Committee of the Faculty of Medicine of the Goethe University Frankfurt. The patients/participants provided their written informed consent to participate in this study.

## Author Contributions

SD, AM, and FS were involved in the design of the study. SD, MW, and JH carried out all data analysis. SD, MW, JH, AM, and FS were involved in the interpretation and discussion of the results. SD took the lead in writing the manuscript. All authors provided critical feedback and contributed to the final manuscript.

## Conflict of Interest

The authors declare that the research was conducted in the absence of any commercial or financial relationships that could be construed as a potential conflict of interest.

## Footnotes

1 www.spm1d.org

## Abbreviations

BMI: body mass index
CF: contact forces
KAM: knee adduction moment
LKCF: lateral tibiofemoral contact force
MKAM: knee adduction moment around the medial condyle
MKCF: medial tibiofemoral contact force
OA: osteoarthritis
SPM: statistical parametric mapping
THR: total hip replacement.
