# Supplementary material for: Knee Load Distribution in Hip Osteoarthritis Patients After Total Hip Replacement
Source: Front Bioeng Biotechnol. 2020 Sep 21;8:578030. doi: 10.3389/fbioe.2020.578030 (PMC7534409; doi:10.3389/fbioe.2020.578030)
Supplement: Supplementary file 1 [file Presentation_1.pdf]

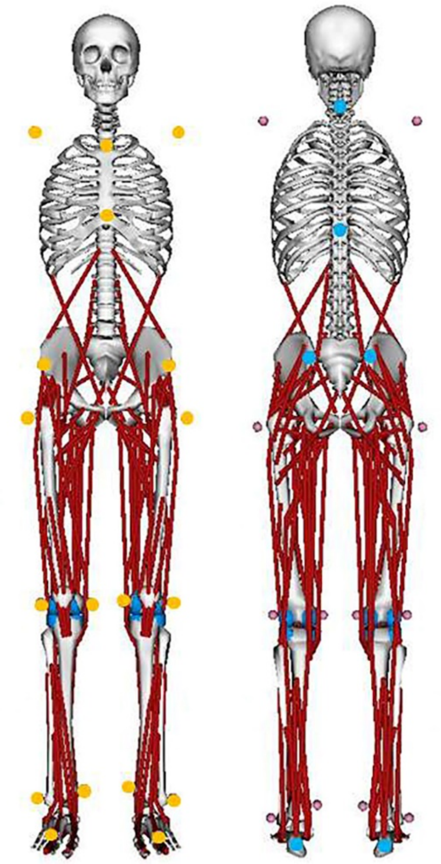

Marker set

Front (orange dots)

- right/left acromioclavicular joint
- incisura jugularis
- xiphoid process
- right/left anterior superior iliac spine
- right/left trochanter major
- right/left lateral femoral condyle
- right/left medial femoral condyle
- right/left lateral malleolus
- right/left medial malleolus
- right/left 2nd metatarsal head

Back (blue dots)

- processus spinosus of the 7th cervical and 10th thoracic vertebra
- right/left posterior superior iliac spine
- right/left heel at the aspect of the achilles tendon insertion (at the same height as the toe markers)

Supplementary Figure 1: Used marker set with reference to the anatomical landmarks.

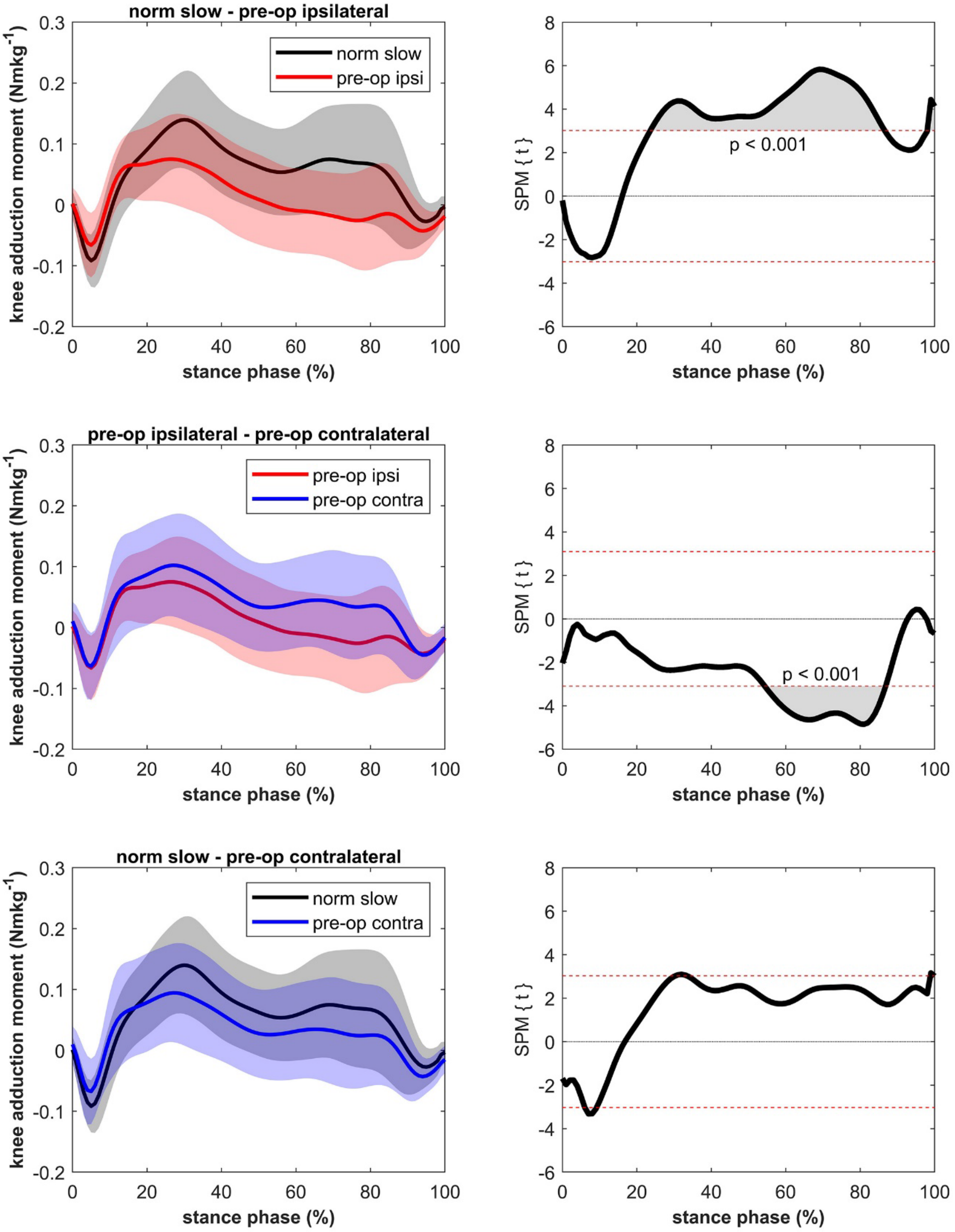

Supplementary Figure 2: Curves of the preoperative external knee adduction moments (bands represent standard deviation) on the left side and the SPM *t*-test results on the right side; comparing controls (norm slow in black) to ipsilateral side of preoperative patients (pre-op ipsi in red) at the top, ipsilateral side of preoperative patients (pre-op ipsi in red) to the contralateral side (pre-op contra in blue) in the middle and controls (norm slow in black) to the contralateral side of the preoperative patients (pre-op contra in blue) at the bottom.

When the SPM *t*-values exceed the critical threshold (dashed horizontal line), differences are significant. Where significant differences are found for more than 4 successive time points (4% of the stance phase of the gait cycle), areas are shaded grey and *p*-values are reported.

Knee adduction moments are normalized to body mass and expressed in $Nmkg^{-1}$.

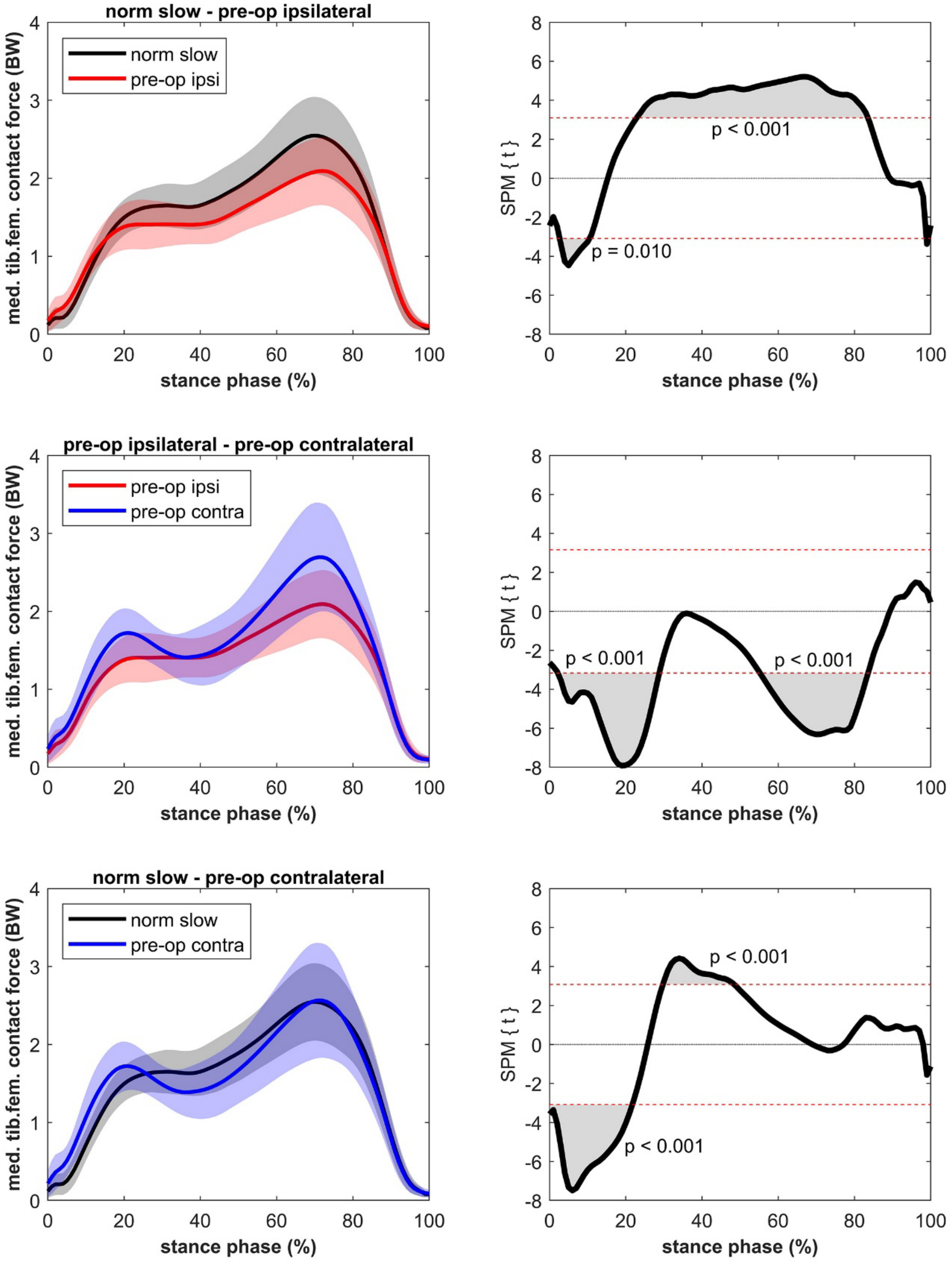

Supplementary Figure 3: Curves of the preoperative tibiofemoral contact force on the medial epicondyle (bands represent standard deviation) on the left side and the SPM *t*-test results on the right side; comparing controls (norm slow in black) to ipsilateral side of preoperative patients (pre-op ipsi in red) at the top, ipsilateral side of preoperative patients (pre-op ipsi in red) to the contralateral side (pre-op contra in blue) in the middle and controls (norm slow in black) to the contralateral side of the preoperative patients (pre-op contra in blue) at the bottom.
When the SPM *t*-values exceed the critical threshold (dashed horizontal line), differences are significant. Where significant differences are found for more than 4 successive time points (4% of the stance phase of the gait cycle), areas are shaded grey and *p*-values are reported.
Contact forces are normalized to body weight (BW).

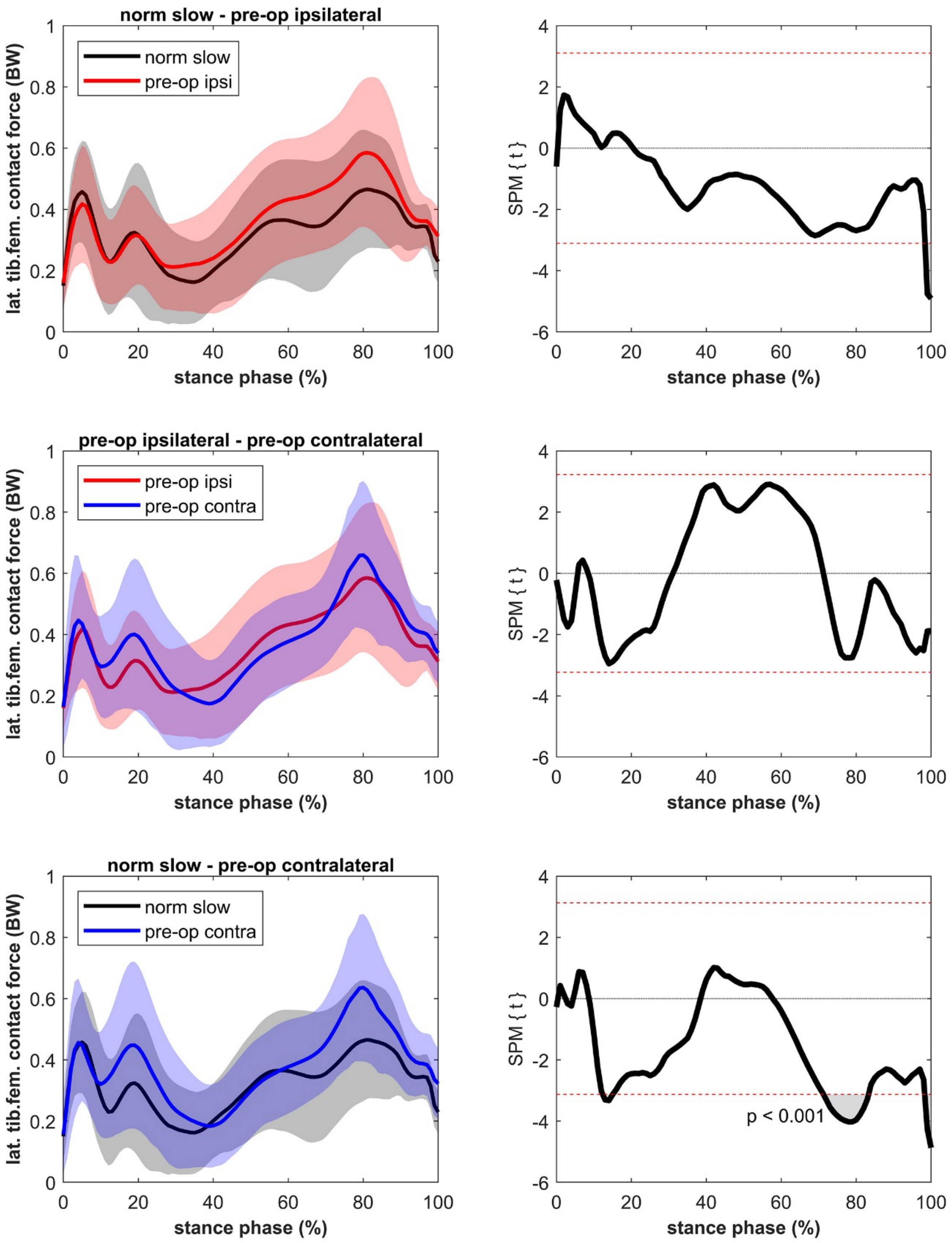

Supplementary Figure 4: Curves of the preoperative tibiofemoral contact force on the lateral epicondyle (bands represent standard deviation) on the left side and the SPM *t*-test results on the right side; comparing controls (norm slow in black) to ipsilateral side of preoperative patients (pre-op ipsi in red) at the top, ipsilateral side of preoperative patients (pre-op ipsi in red) to the contralateral side (pre-op contra in blue) in the middle and controls (norm slow in black) to the contralateral side of the preoperative patients (pre-op contra in blue) at the bottom.
When the SPM *t*-values exceed the critical threshold (dashed horizontal line), differences are significant. Where significant differences are found for more than 4 successive time points (4% of the stance phase of the gait cycle), areas are shaded grey and *p*-values are reported.
Contact forces are normalized to body weight (BW).

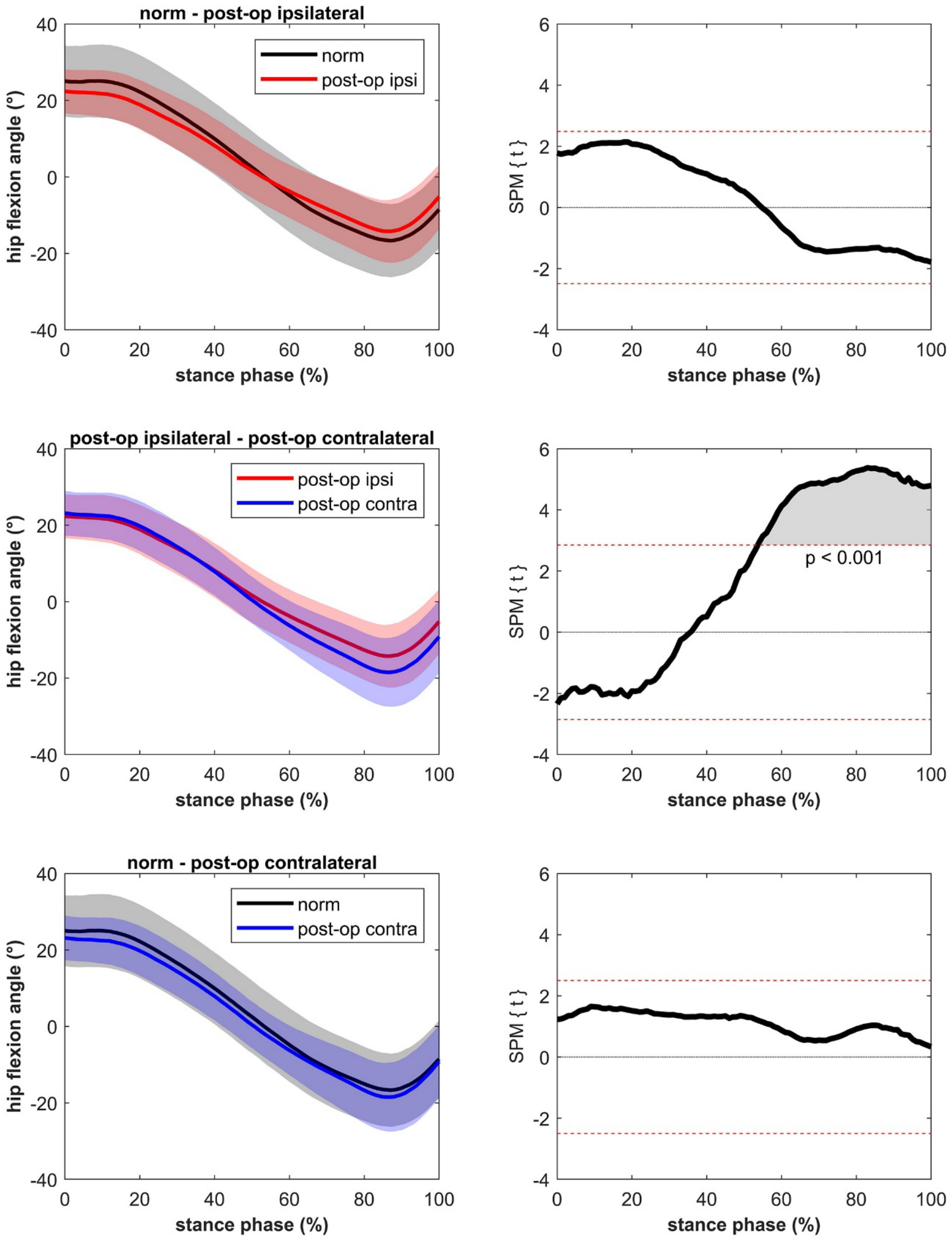

Supplementary Figure 5: Kinematic curves of the postoperative hip flexion/extension (bands represent standard deviation) on the left side and the SPM *t*-test results on the right side; comparing controls (norm in black) to ipsilateral side of postoperative patients (post-op ipsi in red) at the top, ipsilateral side of postoperative patients (post-op ipsi in red) to the contralateral side (post-op contra in blue) in the middle and controls (norm in black) to the contralateral side of the postoperative patients (post-op contra in blue) at the bottom.

When the SPM *t*-values exceed the critical threshold (dashed horizontal line), differences are significant. Where significant differences are found for more than 4 successive time points (4% of the stance phase of the gait cycle), areas are shaded grey and *p*-values are reported.

All angles are expressed in degrees: hip flexion is positive, whereas hip extension is negative.

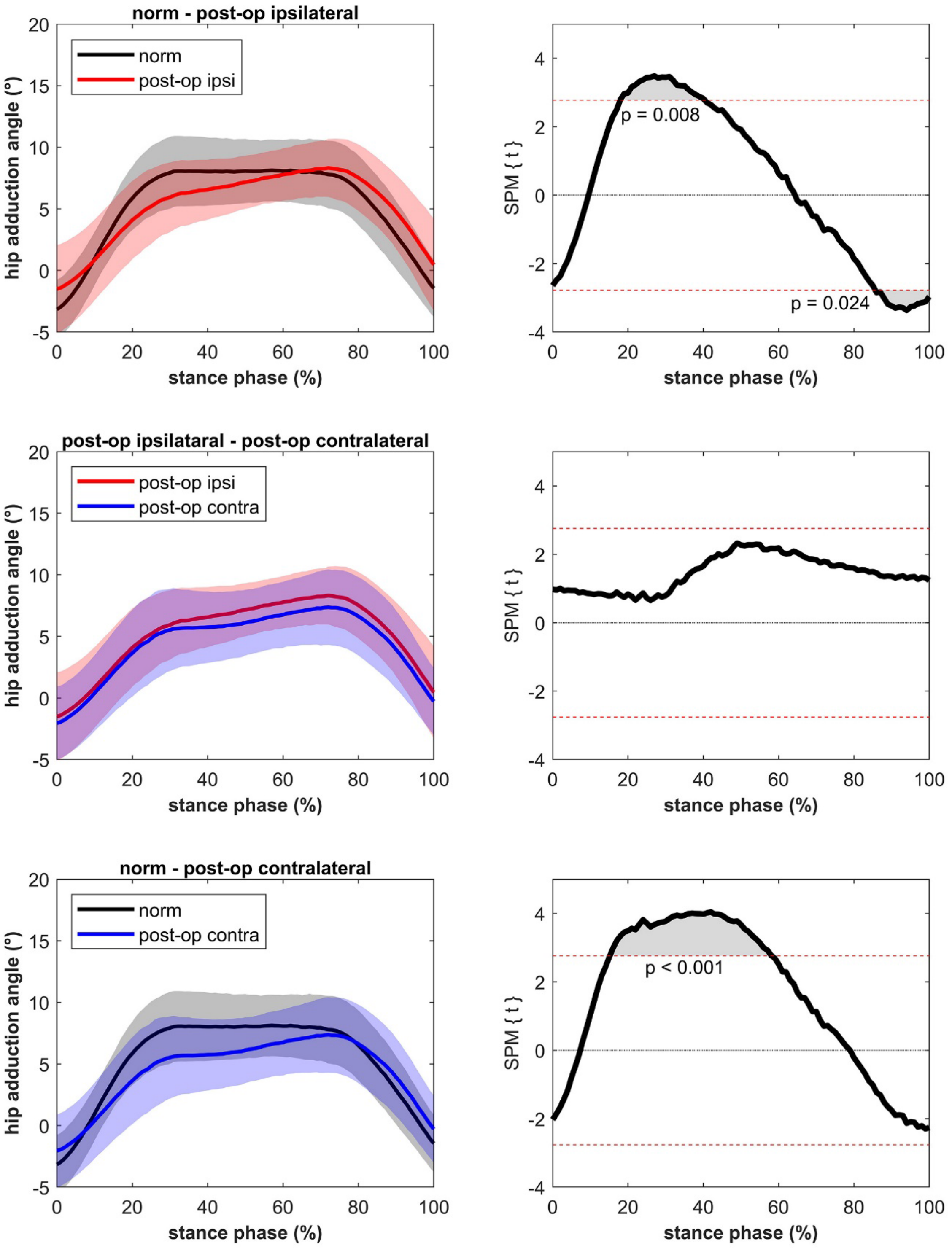

Supplementary Figure 6: Kinematic curves of the postoperative hip adduction/abduction (bands represent standard deviation) on the left side and the SPM *t*-test results on the right side; comparing controls (norm in black) to ipsilateral side of postoperative patients (post-op ipsi in red) at the top, ipsilateral side of postoperative patients (post-op ipsi in red) to the contralateral side (post-op contra in blue) in the middle and controls (norm in black) to the contralateral side of the postoperative patients (post-op contra in blue) at the bottom.

When the SPM *t*-values exceed the critical threshold (dashed horizontal line), differences are significant. Where significant differences are found for more than 4 successive time points (4% of the stance phase of the gait cycle), areas are shaded grey and *p*-values are reported.

All angles are expressed in degrees: hip adduction is positive, whereas hip abduction is negative.

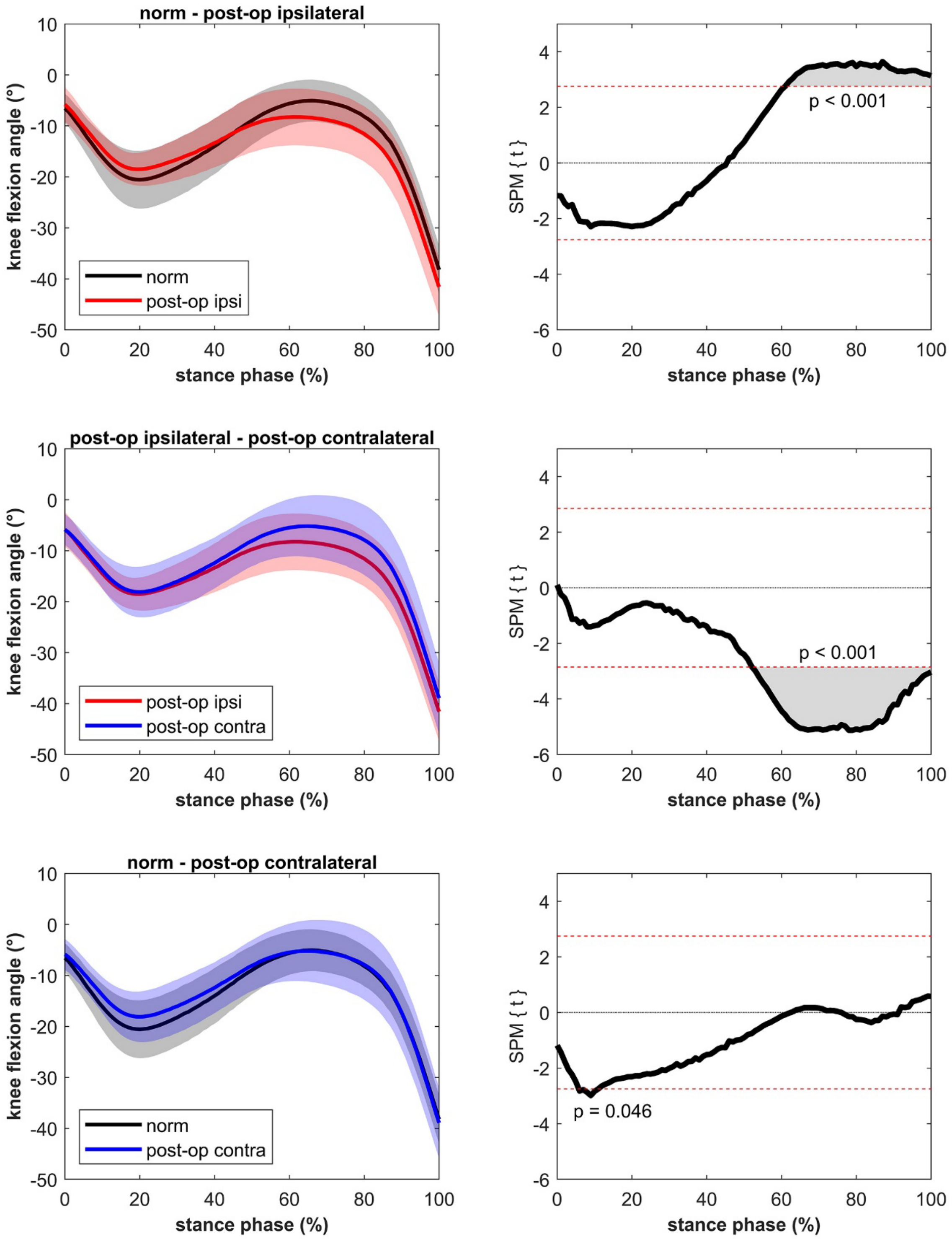

Supplementary Figure 7: Kinematic curves of the postoperative knee flexion/extension (bands represent standard deviation) on the left side and the SPM *t*-test results on the right side; comparing controls (norm in black) to ipsilateral side of postoperative patients (post-op ipsi in red) at the top, ipsilateral side of postoperative patients (post-op ipsi in red) to the contralateral side (post-op contra in blue) in the middle and controls (norm in black) to the contralateral side of the postoperative patients (post-op contra in blue) at the bottom.

When the SPM *t*-values exceed the critical threshold (dashed horizontal line), differences are significant. Where significant differences are found for more than 4 successive time points (4% of the stance phase of the gait cycle), areas are shaded grey and *p*-values are reported.

All angles are expressed in degrees: knee extension is positive, whereas knee flexion is negative.
